# Cancer Association in Patients with Immune-Mediated Inflammatory Diseases: A Five-Year Nationwide Italian Cohort Study

**DOI:** 10.3390/cancers18061027

**Published:** 2026-03-23

**Authors:** Barbara Giordani, Luigi Pirtoli, Guido Putignano, Antonio Giordano, Daniela Marotto, Giovanni Baglio

**Affiliations:** 1 Italian National Agency for Regional Healthcare Services (AGENAS), 00187 Rome, Italy; baglio@agenas.it; 2 Sbarro Institute, College of Science and Technology’s Biotechnology, Temple University, Philadelphia, PA 19122, USA; luigi.pirtoli@shro.org (L.P.); antonio.giordano@temple.edu (A.G.); 3 Department of Medical Biotechnology, University of Siena, 53100 Siena, Italy; 4 bioERGOtech, 74123 Taranto, Italy; guido.putignano@bioergotech.org; 5 Azienda Socio-Sanitaria Locale della Gallura, 07026 Olbia, Italy; daniela.marotto@aslgallura.it

**Keywords:** Immune-mediated inflammatory diseases (IMID), rheumatoid arthritis, diffuse diseases of the connective tissue, cancer, malignancy, hospital-based cohort study

## Abstract

**Background**: Cancer represents a major concern in rheumatology. Epidemiological studies have demonstrated associations between rheumatic conditions and increased cancer risk at specific sites. However, comprehensive Italian epidemiological data on this association remain lacking. **Methods:** We conducted a nationwide retrospective cohort study using Italian Hospital Discharge Records from patients hospitalized between 1 January and 31 December 2018, with follow-up until 31 December 2023. Adult patients with Immune-mediated inflammatory diseases (IMID), including rheumatoid arthritis (RA) and diffuse diseases of connective tissue (DDCT), were the exposed group. The unexposed group included patients with primary diagnoses of injury and poisoning, with no IMID history. We used multivariable logistic regression models to assess cancer association, adjusting for demographics, geography, time of follow-up and comorbidities. **Results:** We included 54,896 IMID patients (exposed) and 301,126 unexposed. During the five-year follow-up, the IMID group was significantly associated with cancer (adjusted OR 1.32, 95% CI 1.27–1.38; *p* < 0.001). The OR_adj_ decreased over time, from 1.83 (*p* < 0.001) in year one to 1.20 (*p* < 0.001) from the fifth year onward. Significant association with lung cancer (OR_adj_ 1.74; *p* < 0.001), leukemia and lymphoma (OR_adj_ 1.98; *p* < 0.001), bladder (OR_adj_ 1.48; *p* < 0.001) and melanoma (OR_adj_ 1.48; *p* = 0.009) were observed in the IMID group. DDCT patients showed an overall higher cancer risk (OR_adj_ 1.53, *p* < 0.001) than RA patients (OR_adj_ 1.20, *p* < 0.001). **Conclusions:** This first nationwide Italian study confirms an association with cancer in IMID patients, with the highest association in the first year post-diagnosis. The temporal decrease in cancer risk generates the hypothesis that inflammation, rather than medication, may be the primary driver. These findings support enhanced cancer surveillance in IMID patients, particularly during the first year after diagnosis.

## Simple Summary

This study provides the first nationwide Italian evidence on cancer risk in Immune-mediated inflammatory diseases (IMID) encompassing 356,022 patients with five-year follow-up. Our broader inclusion criteria beyond traditional rheumatoid arthritis populations provide insights into diverse connective tissue conditions. We found that association with cancer onset is highest immediately after IMID diagnosis; the temporal decline in cancer risk over subsequent years supports the hypothesis of inflammation-driven rather than treatment-related mechanisms, reinforcing the potential protective effects of early anti-inflammatory therapy. The consistent findings across international populations suggest that enhanced cancer surveillance protocols should be implemented for IMID patients, particularly during the first year after diagnosis. The temporal pattern of risk reduction supports early anti-inflammatory treatment strategies. Future research should focus on identifying specific inflammatory biomarkers that predict cancer risk.

## 1. Introduction

In recent decades, cancer research initially focused on six core hallmarks of tumor cells: self-sufficiency of growth signals, insensitivity to anti-growth signals, escaping from apoptosis, unregulated proliferation potential, enhanced angiogenesis, and metastasis [1]. More recently, Weinberg recognized that this approach neglected the domain of tumor microenvironment (TME), including immunity and inflammation [2]. The most recent update of Hanahan’s “hallmarks of cancer” recognized the TME interaction with cancer cells, expanding the framework to fourteen hallmarks, which vary across malignancies and even subtypes [3]. Despite this, the role of TME in cancer origin and progression remains incompletely understood due to its complexity. In fact, TME can be considered as the connective tissue proper of neoplasms, through which dysfunctional molecules and signal pathways can influence cancer initiation and progression, as in the case of inflammation and immunity determinants, representing the crossroad between connective tissue disorders and cancer. This insight has also paved the way for new treatment strategies, such as immunotherapy of cancer that, in turn, induces immune adverse effects including rheumatological events [4].

Advances in multiplexed imaging, single-cell RNA sequencing, spatial transcriptomics, and AI-based platforms enable precise characterization of the tumor immune microenvironment [5]. These technologies are particularly valuable for dissecting mechanisms linking chronic inflammation, autoimmunity, and cancer development in connective tissue diseases, where immune dysregulation drives pathogenesis [1].

The rheumatology community has recently explored the association between rheumatological diseases (characterized in most cases by autoimmunity and inflammation) and the risk of developing cancer, establishing the field of “oncorheumatology”, which addresses both cancer in systemic autoimmune rheumatologic disease (SARD) patients and musculoskeletal pathology in cancer not directly related to neoplastic diffusion [6]. This approach is further supported by solid evidence, although it has yet to gain widespread acknowledgment in oncology or rheumatology scientific communities [7,8].

The term “connective tissue disorders” is infrequently used in scientific literature and more commonly appears in general or educational contexts, but the International Classification of Diseases, 9th revision Clinical Modification, adopted by the Italian National Healthcare Service [9], is suitable for the research method adopted in this paper, as it allows case retrieval in hospital records [10].

The primary aim of this study is to investigate the association between Immune-Mediated Inflammatory Diseases (IMID) and first cancer occurrence, both overall and by specific cancer sites. A secondary aim is to assess the potential association with cancer risk in two subgroups of patients—those with rheumatoid arthritis (RA) and those with diffuse connective tissue diseases (DDCT)—drawing on the available oncology and rheumatology literature, which is predominantly focused on these diseases.

## 2. Materials and Methods

### 2.1. Study Design and Data Source

We conducted a nationwide retrospective cohort study using Hospital Discharge Records (HDRs) of the National Healthcare Service, updated to 31 December 2023, and provided by approximately 1100 public and private hospitals throughout Italy. HDR data are routinely collected by the Italian Ministry of Health and contain patient demographic information (including gender and age), admission and discharge dates, up to six discharge diagnoses (ICD-9-CM codes), eleven medical procedures or surgical interventions, and status at discharge (alive, dead, or transferred to another hospital). HDRs were subsequently linked with the National Tax Registry to determine vital status after hospitalization. The HDRs provide nationwide coverage of hospital admissions, and no missing data were reported for the key variables used in the study.

### 2.2. Study Population and Follow-Up

The analysis included patients hospitalized between 1 January and 31 December 2018 (index year) to investigate the association between IMID and cancer occurrence through 31 December 2023, providing a follow-up period of around five years. We acknowledge that our study cohort encompasses heterogeneous conditions with distinct pathophysiology, including true connective tissue diseases (systemic lupus erythematosus, systemic sclerosis), inflammatory arthritides (rheumatoid arthritis, spondyloarthritis), and crystal arthropathies (gout). Additionally, some included codes represent non-specific conditions (e.g., unspecified rheumatism, myalgia) or non-autoimmune processes (e.g., infectious arthropathies). Due to the constraints of administrative database methodology using ICD-9-CM codes, we pragmatically grouped these under the umbrella term of Immune-mediated Inflammatory Diseases (IMID), as detailed in Table 1, while recognizing this represents a methodological rather than clinically optimal classification approach.

### 2.3. Exposed Group (IMID Patients)

The exposed group comprised adult patients (aged ≥ 18 years) discharged from ordinary wards or day-hospital services, residing in Italy, with at least one of the following diagnosis codes (recorded in the index year or in the previous five years):

### 2.4. Unexposed Group (No IMID Patients)

The unexposed group included adult patients (aged ≥18 years) discharged from ordinary wards or day-hospital services, with a primary diagnosis of injury and poisoning (ICD-9-CM codes: 800-999) and no previous history of IMID in the past five years, in the index year and in the follow-up period.

#### Exclusion Criteria

We excluded all patients who died during hospitalization or had a previous history of malignancy (ICD-9-CM codes: 140-208, and V10) from both the exposed and unexposed groups.

### 2.5. Outcome Definition

The primary outcome, identified from any diagnosis position in HDRs, was the first occurrence of any cancer during the follow-up period, identified through ICD-9-CM codes 140-208. Secondary outcomes included site-specific cancers, with particular focus on malignancies previously reported to be associated with IMIDs in the literature.

### 2.6. Statistical Analysis

Sociodemographic variables (age and gender) were included in the analysis. Patients were also classified according to their geographic area of residence within the three main Italian geographical regions (North, Center, South and Islands).

The crude 5-year cumulative incidence was calculated as the proportion of patients with a cancer diagnosis divided by the total number of patients in each group.

We assessed the association between IMID and cancer occurrence (overall and site-specific) using multivariable logistic regression models [11]. The follow-up time started from the admission date (of the index year) to the occurrence date of the first cancer diagnosis or to the end of the study period (31 December 2023). Patients who died during the follow-up were censored at the time of death. The analysis was adjusted for age, gender, geographic area, time of follow-up and comorbidities (binary variable), recorded in the index year and in the previous five years, to compare the exposed and unexposed groups. Adjustment variables included: hypertension, obesity, diabetes, ischemic heart disease and other cardiac conditions, cerebrovascular and encephalic circulatory disorders, chronic kidney disease, chronic inflammatory bowel diseases, diseases of pulmonary circulation, chronic obstructive pulmonary disease and chronic respiratory failure, other chronic respiratory diseases, and moderate to severe liver disease.

We performed two additional multivariable logistic regression analyses separately for two subgroups of exposed patients: those affected by RA (code ‘714’) and those with DDCT (code ‘710’).

All analyses were performed using SAS Studio (version 3.81; SAS Institute; Enterprise edition).

## 3. Results

### 3.1. Baseline Characteristics

We identified 54,896 patients with Immune-mediated inflammatory diseases (IMID; exposed group) and 301,126 patients without IMID (unexposed group) out of those discharged from Italian public and private hospitals. The IMID group had a higher proportion of women than the unexposed group (73.6% vs. 50.8%; Table 2). Approximately half of the patients in both groups were aged 65 years or older. Patients with IMID had substantially higher rates of comorbidities across all measured conditions, with the most prevalent being hypertension (27.0%), cerebrovascular and arterial diseases (23.4%), and chronic rheumatic heart disease (12.2%), compared with 9.0%, 5.9%, and 3.2%, respectively, in the unexposed group.

### 3.2. Overall Cancer Risk

During the five-year follow-up period, patients with IMID were 32% more likely to develop cancer compared with unexposed patients; this association remained significant after adjusting for demographics, geographical region, and comorbidities (OR_adj_ 1.32, 95% CI 1.27–1.38; *p* < 0.001; Table 3).

### 3.3. Cancer Risk over Time

The cancer risk in IMID patients was not constant over time. Risk was highest by the first year after IMID diagnosis (OR_adj_ 1.83, 95% CI 1.61–2.08; *p* < 0.001) and decreased progressively in subsequent years: second year (OR_adj_ 1.53, 95% CI 1.37–1.69; *p* < 0.001), third year (OR_adj_ 1.40, 95% CI 1.25–1.56; *p* < 0.001), fourth year (OR_adj_ 1.37, 95% CI 1.22–1.53; *p* < 0.001), and fifth year onwards (OR_adj_ 1.20, 95% CI 1.15–1.30; *p* < 0.001).

### 3.4. IMID Subtypes and Site-Specific Cancers

**Figure 1:**
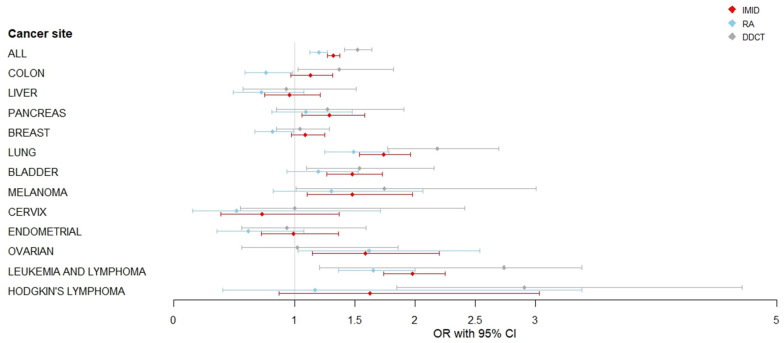
Risk of cancer by site and by different subgroups of Immune-mediated Inflammatory Diseases. Forest plot showing adjusted odds ratios (OR) and 95% confidence intervals for site-specific cancers in patients with Immune-mediated inflammatory diseases compared with unexposed groups. IMID = Immune-mediated inflammatory diseases. RA = rheumatoid arthritis. DDCT = diffuse diseases of the connective tissue. ORs were adjusted for gender (if applicable), age, Italian geographical area of residence, time of follow-up and comorbidities. The vertical dashed line represents OR = 1.0 (no association). Squares represent point estimates; horizontal lines represent 95% confidence intervals.

When we analyzed specific IMID subtypes, patients with diffuse connective tissue diseases (DDCT) showed an overall association with a subsequent cancer diagnosis (OR_adj_ = 1.53, 95% CI 1.42–1.64; *p* < 0.001) than those with rheumatoid arthritis (OR_adj_ 1.20, 95% CI 1.12–1.28; *p* < 0.001; Figure 1). All IMID subtypes showed significantly association with lung cancer risk, in DDCT patients (OR_adj_ 2.18, 95% CI 1.77–2.69; *p* < 0.001), in overall IMID (OR_adj_ 1.74, 95% CI 1.54–1.96; *p* < 0.001) and RA patients (OR_adj_ 1.49, 95% CI 1.25–1.78; *p* < 0.001). Association with bladder cancer was found in both the IMID (OR_adj_ = 1.48, 95% CI 1.27–1.73; *p* < 0.001) and DDCT groups (OR_adj_ = 1.54, 95% CI 1.10–2.16; *p* = 0.012). The association with leukemia and lymphoma was more than doubled in DDCT patients (OR_adj_ 2.74, 95% CI 2.21–3.38; *p* < 0.001), with significant but lower increases observed in the overall IMID group (OR_adj_ 1.98, 95% CI 1.74–2.25; *p* < 0.001) and in RA patients (OR_adj_ 1.65, 95% CI 1.37–2.00; *p* < 0.001). We also observed an association with Hodgkin’s lymphoma and colon cancer in the DDCT group and with melanoma in the IMID group (OR_adj_ = 1.48, 95% CI 1.10–1.98; *p* = 0.009), mainly attributable to the IMID men group (OR_men_ = 1.59, 95% CI 1.05–2.40; *p* = 0.029). We observed weak evidence of decreased risk of colon (OR_adj_ = 0.76, 95% CI 0.59–0.99; *p* = 0.037) and breast (OR_adj_= 0.82, 95% CI 0.67–0.99; *p* = 0.033) cancers in RA patients compared to the unexposed.

Among IMID women, we observed strong associations with Hodgkin’s lymphoma (OR_adj_ = 3.93, 95% CI 1.77–6.76; *p* = 0.001), bladder cancer (OR_adj_ = 1.97, 95% CI 1.52–2.55; *p* < 0.001), and ovarian cancer (OR_adj_ = 1.59, 95% CI 1.15–2.20; *p* = 0.005). Women with RA were 62% more likely to develop ovarian cancer than unexposed (OR_adj_ = 1.62, 95% CI 1.03–2.54; *p* = 0.037), and DDCT was associated with more than a five-fold association with Hodgkin’s lymphoma in women (OR_adj_ = 5.68, 95% CI 1.85–15.42; *p* = 0.002).

No significant associations were observed between any of the IMID groups and liver cancer (OR_adj_ = 0.96, 95% CI 0.75–1.21; *p* = 0.712). Similarly, we found no association with hormone-related malignancies, including breast (OR_adj_ = 1.10, 95% CI 0.98–1.25; *p* = 0.117), cervical (OR_adj_ = 0.73, 95% CI 0.39–1.37; *p* = 0.328), and endometrial cancers (OR_adj_ = 0.99, 95% CI 0.72–1.36; *p* = 0.968).

## 4. Discussion

The association between Immune-mediated inflammatory diseases (IMID) and cancer is changing our understanding of inflammatory disease pathogenesis. Since the late 1970s, growing evidence has linked Immune-mediated inflammatory diseases to haematologic and solid cancers, notably rheumatoid arthritis to lymphomas [12]. This nationwide Italian study provides the first comprehensive epidemiological evidence of 32% higher overall cancer risk of patients with IMIDs over five years of follow-up, highlighting the importance of oncorheumatology and cancer surveillance in IMID management.

Given the observational and administrative nature of data, the results prompted us to generate hypotheses about important mechanistic insights into the temporal pattern of cancer risk observed in our cohort. The highest association was observed during the first year following IMID diagnosis (OR_adj_ = 1.83), with progressive decline in subsequent years, reaching a more modest increase by the fifth year onward (OR_adj_ = 1.20). This pattern suggests that systemic inflammation, rather than immunosuppressive treatments, drives the cancer-IMID association. Early inflammatory states may create a tumor-promoting microenvironment through multiple pathways, including enhanced angiogenesis, immune surveillance disruption, and cellular DNA damage. The temporal decline in risk supports the hypothesis that effective anti-inflammatory treatment may provide oncological benefits, offering additional justification for early disease management. The results may also lead to the hypothesis that increased medical surveillance following IMID diagnosis may facilitate earlier cancer detection; alternatively, subclinical malignancies present at IMID onset might become clinically apparent, or early inflammatory states could accelerate tumor development.

Even after adjusting for IMID and other covariates, older age groups show a significantly higher risk of developing cancer compared with younger individuals. As described by Maier et al. [13], chronic inflammation in Immune-mediated diseases creates environments conducive to oncogenesis. The convergence of aging-related immunosenescence and persistent inflammation may critically impair immune surveillance, reinforcing the need for age-stratified cancer surveillance protocols [2].

Evidence from this paper closely aligns with recent international studies while providing unique insights into the IMID spectrum. Previous meta-analyses addressing rheumatoid arthritis have reported pooled standardized incidence ratios for all-site malignancy risk of 1.09 [14,15], consistent with our findings. However, this co-occurrence can vary quantitatively according to cancer and Immune-mediated inflammatory disease types, influenced by variable factors such as personal medical history, ethnicity, lifestyle factors, immunosuppressive therapies, and classification criteria [16]. Our broader inclusion criteria encompassing diverse IMIDs revealed important disease-specific variations. Patients with diffuse connective tissue diseases showed significantly higher cancer association (OR_adj_ = 1.53) than those with rheumatoid arthritis (OR_adj_ = 1.20), suggesting that disease heterogeneity within the IMID spectrum influences malignancy potential.

Our findings must be interpreted in the context of well-established disease-specific cancer associations. Dermatomyositis patients face particularly elevated malignancy risks (2–7 fold increase), often temporally related to myositis onset and associated with specific autoantibodies. Similarly, systemic sclerosis patients demonstrate increased lung cancer risks, particularly those with anti-RNA polymerase III antibodies. While our pooled analysis provides population-level insights, the heterogeneous nature of our cohort prevents the detection of these clinically crucial disease-specific patterns.

Evidence from scientific literature highlights particularly striking associations with lymphoid malignancies and lung cancer, consistent with established inflammatory pathways in oncogenesis. Simon et al. previously reported increased risks of lymphoma, particularly diffuse large B-cell lymphoma, and lung cancer in patients with rheumatoid arthritis compared with the general population, while observing decreased risks of colorectal and breast cancers [15]. Our findings extend these observations across the broader IMID spectrum. Strong association with leukemia and lymphoma in DDCT patients (OR_adj_ = 2.74) likely reflects the chronic B-cell activation and dysregulated immune responses characteristic of these conditions.

Although Wu et al. observed a higher incidence of lung cancer in RA patients, their Mendelian randomisation study did not support a genetically mediated causal link [17]. We found a strong association with higher lung cancer risk across all IMID subtypes, suggesting that acquired inflammatory processes rather than genetic predisposition may drive this association. These findings underscore the need for organ-specific surveillance strategies tailored to the underlying IMID diagnosis.

Results on IMID are also consistent with prior studies on systemic lupus erythematosus showing a 1.14-fold increase in overall cancer risk, with 4-fold elevation for non-Hodgkin lymphoma, though hormonal factors or SLE-related antibodies may drive decreased risks of breast, ovarian, and endometrial cancers [18,19].

For other IMIDs, malignancy data vary considerably. Spondylarthritis studies show conflicting results, with some highlighting only small overall increases or no association with cancer risk [20,21].

However, multiple authors have emphasized generally increased skin cancer risk across systemic autoimmune rheumatic diseases [14,18,19]. Significant correlations between breast cancer and psoriatic arthritis have been observed [22,23,24], while primary Sjögren’s syndrome associates with increased mucosa-associated lymphoid tissue lymphoma and solid tumor risks [25,26]. Danish studies demonstrate 2.5-fold increased hematological malignancy risk in scleroderma patients, with higher risks in males, those with RNA polymerase III autoantibodies, and elderly-onset disease [27,28]. Comprehensive reviews by Cappelli et al. addressed malignancy risks across systemic autoimmune rheumatic diseases, including idiopathic inflammatory myopathies, anti-neutrophil cytoplasmic antibody vasculitis, and polymyalgia rheumatica/giant cell arteritis 6. Recently, French authors proposed detailed screening recommendations for patients with systemic autoimmune rheumatic diseases, including those receiving immunosuppressive treatments [29].

Comparison with the detailed French nationwide study by Beydon et al. reveals similarities for most of the tumor sites analyzed and some important differences [30].

For breast and colon cancer, findings from both studies suggest a potentially protective association. One possible hypothesis is that, for breast cancer, differences in endogenous hormonal exposure—such as earlier menopause and younger age at first pregnancy, which are associated with increased RA risk but lower breast cancer risk—may partly explain the lower incidence [30]. Meta-analysis has shown that long-term aspirin and non-aspirin nonsteroidal anti-inflammatory drugs (NSAIDs) use is linked to a decreased incidence of recurrent colorectal adenomas [31].

However, while an increased risk of cervical cancer was reported in their cohort, no association was found in ours, likely reflecting differences between the nationwide population-based and hospital-based approaches.

The use of administrative ICD-9-CM codes encompassed a heterogeneous spectrum of inflammatory and musculoskeletal conditions, including non-autoimmune processes (infectious and crystal arthropathies) and non-specific diagnostic categories (unspecified rheumatism, myalgia). While this broad inclusion reflects real-world clinical coding practices and provides population-level insights across inflammatory conditions, it may have attenuated cancer risk estimates for well-defined autoimmune diseases through misclassification bias.

Our separate analyses of DDCT and rheumatoid arthritis, representing more homogeneous autoimmune populations, provide clinically relevant estimates with reduced misclassification.

Several additional important methodological aspects warrant consideration when interpreting our findings.

The absence of precise disease onset timing prevents definitive assessment of temporal relationships between IMID development and malignancy; the development of cancer may involve a substantial latency period, and therefore, the observed associations may reflect processes that began before the recorded IMID diagnosis. Moreover, missing inflammatory markers for enhancing early detection and personalized treatment strategies [32] and disease activity measures limit the evaluation of how disease severity influences cancer risk. Important determinants of cancer risk were not available in the HDRs, including smoking status, alcohol consumption, and socioeconomic status. This may have introduced residual confounding and could have influenced the observed associations. Additionally, the lack of data on specific medications, including immunosuppressive and anti-inflammatory therapies, biologics, corticosteroids, or disease activity, which are critical modifiers of cancer risk in IMID populations, represents potential unmeasured confounding, though our temporal risk patterns suggest inflammation-driven rather than treatment-related mechanisms. Despite these constraints, our substantial sample size of over 356,000 patients provides robust statistical power and represents the most comprehensive investigation of IMID-cancer associations conducted in Italy.

This nationwide study confirms cancer as a significant comorbidity in IMID patients, highlighting temporal risk patterns that inform disease pathogenesis and clinical management. The highest risk in the first year after diagnosis has implications for clinical practice, supporting cancer surveillance protocols when the risks peak and tailored strategies based on specific autoimmune diagnoses, as current guidelines recommend. Screening should be individualized based on the underlying autoimmune condition and established disease-specific cancer associations, such as lymphomas, lung cancer, and gender-specific malignancies, including Hodgkin’s lymphoma, bladder cancer, and ovarian cancer, identified in this paper.

The temporal risk pattern supports early anti-inflammatory treatment for both disease control and potential cancer prevention, while suggesting that conventional and targeted disease-modifying anti-rheumatic drugs may exert protective effects through inflammatory pathway modulation.

## 5. Conclusions

Our comprehensive approach covering diverse IMIDs provides valuable insights into the complex relationship between autoimmune diseases and cancer across the connective tissue disease spectrum. The findings generate a hypothesis of a potential bidirectional association between chronic inflammation and tumorigenesis and underscore the importance of multidisciplinary care approaches integrating rheumatological and oncological expertise. Future research should focus on identifying predictive biomarkers for individualized cancer risk assessment, examining the impact of specific therapeutic agents on malignancy incidence, and clarifying biological mechanisms underlying autoimmunity-cancer links. These findings provide epidemiological evidence to develop guidelines improving outcomes for patients with these complex, interconnected conditions, ultimately supporting the evolution of personalized medicine approaches in the emerging field of oncorheumatology.

## Figures and Tables

**Table 1:** International Classification of Diseases, 9th revision Clinical Modification (ICD-9-CM).

ICD Code	Disease Description
**710**	**Diffuse diseases of the connective tissue (DDCT)**
710.0	Systemic Lupus Erythematosus
710.1	Systemic Sclerosis
710.2	Sjögren’s syndrome
710.3	Dermatomyositis
710.4	Polymyositis
710.5	Eosinophilia Myalgia Syndrome
710.8	Other specified diffuse diseases of connective tissue
710.9	Other unspecified diffuse diseases of connective tissue
**711**	**Arthropathy associated with infections**
**712**	**Crystal arthropathies**
**713**	**Arthropathy associated with other disorders classified elsewhere**
**714**	**Rheumatoid arthritis and other inflammatory polyarthropathies (RA)**
**720**	**Ankylosing spondylitis and other inflammatory spondylopathies**
729.0	Rheumatism, unspecified and fibrositis
729.1	Myalgia and myositis, unspecified
716.3	Climacteric arthritis

**Table 2:** Baseline demographic and clinical characteristics of patients with and without connective tissue diseases (IMID).

Characteristics	Presence of IMID (Exposed Group) N = 54,896 (100%)	Absence of IMID (Unexposed Group) N = 301,126 (100%)
**Gender**		
Male	14,504 (26.4)	148,167 (49.2)
Female	40,392 (73.6)	152,959 (50.8)
**Age class**		
18–34	3749 (6.8)	46,825 (15.6)
35–54	13,535 (24.7)	72,405 (24.0)
55–64	10,625 (19.3)	43,180 (14.3)
≥65	26,987 (49.2)	138,716 (46.1)
Italian geographic area *		
North	23,014 (41.9)	138,903 (46.1)
Center	11,361 (20.7)	63,838 (21.2)
South and Islands	20,521 (37.4)	98,385 (32.7)
**Comorbidities**		
Diabetes	5924 (10.8)	11,088 (3.7)
Obesity	3097 (5.6)	3541 (1.2)
Hypertension	14,816 (27.0)	27,062 (9.0)
Ischemic heart disease	5899 (10.7)	12,967 (4.3)
Chronic rheumatic heart disease	6710 (12.2)	9676 (3.2)
Cerebrovascular disease and the arteries, arterioles, and capillaries	12,822 (23.4)	17,650 (5.9)
Chronic kidney disease	5945 (10.8)	9210 (3.1)
Liver disease	2983 (5.4)	3044 (1.0)
Chronic inflammatory bowel disease	1507 (2.7)	668 (0.2)
Diseases of pulmonary circulation	694 (1.3)	861 (0.3)
Respiratory failure	3697 (6.7)	4308 (1.4)
Other diseases of the respiratory system	3247 (5.9)	220 (0.1)

(*) The North includes Valle d’Aosta, Piemonte, Liguria, Lombardia, Trentino-Alto Adige, Friuli-Venezia Giulia, Veneto, and Emilia-Romagna regions. The Center includes the Toscana, Umbria, Marche, and Lazio regions. The South and Islands include Abruzzo, Molise, Campania, Puglia, Basilicata, Calabria, Sicilia, and Sardegna regions.

**Table 3:** Risk of cancer by characteristics of patients in the study population.

Characteristics	No. of Patients	No. of Cancer	Crude 5-Year Cumulative Incidence (%)	Adjusted OR *	95% CI	*p*-Value
**IMID**						
Absent	301,126	14,838	4.9	ref.		
Present	54,896	3766	6.9	1.32	1.27–1.38	<0.001
**Gender**						
Male	162,671	8045	4.9	ref.		
Female	193,351	10,559	5.5	0.70	0.68–0.72	<0.001
**Age class**						
18–34	50,574	247	0.5	ref.		
35–54	85,940	2104	2.4	5.17	4.53–5.90	<0.001
55–64	53,805	3178	5.9	13.39	11.76–15.26	<0.001
≥65	165,703	13,075	7.9	19.57	17.23–22.23	<0.001
**Italian geographic area**						
North	161,917	8933	5.5	ref.		
Center	75,199	4217	5.6	1.04	1.01–1.09	0.027
South and Islands	118,906	5454	4.6	0.86	0.83–0.89	<0.001

OR = Odds Ratio. CI = confidence interval. IMID = Immune-mediated inflammatory diseases. * Also adjusted for comorbidities, including diabetes, obesity, hypertension, ischaemic heart disease, chronic rheumatic heart disease, cerebrovascular disease, diseases of arteries, chronic kidney disease, liver disease, chronic inflammatory bowel disease, diseases of pulmonary circulation, respiratory failure, and other diseases of the respiratory system.

## Data Availability

The data are available from AGENAS with its permission and upon reasonable request, by contacting direzione.ricerca@agenas.it.

## Abbreviations

The following abbreviations are used in this manuscript:

CREI	Italian College of Rheumatology
TME	Tumor microenvironment
IMID	Immune-mediated inflammatory diseases
RA	Rheumatoid arthritis
DDCT	Diffuse diseases of connective tissue
